# Evaluation of the effect of different enamel surface treatments and waiting times on the staining prevention after bleaching

**DOI:** 10.4317/jced.53712

**Published:** 2017-05-01

**Authors:** Débora Monteiro, Allyson Moreira, Tulimar Cornacchia, Cláudia Magalhães

**Affiliations:** 1 DDS, MSc, PhD student, Department of Restorative Dentistry. School of Dentistry, Universidade Federal de Minas Gerais and Professor, Faculdade de Estudos Administrativos, Dentistry, FEAD; 2 DDS, PhD, Professor, Department of Restorative Dentistry. School of Dentistry, Universidade Federal de Minas Gerais; 3 DDS, MSc, PhD, Professor, Department of Restorative Dentistry. School of Dentistry, Universidade Federal de Minas Gerais; 4 DDS, MSc, PhD, Professor, Department of Restorative Dentistry. School of Dentistry, Universidade Federal de Minas Gerais

## Abstract

**Background:**

Bleached dental enamel can be more susceptible to staining than the enamel that has never been bleached, especially right after tooth bleaching. The aim of this study is to evaluate the effect of surface treatments and waiting time prior to contact with dye on bleached enamel staining susceptibility.

**Material and Methods:**

One hundred teeth were bleached with 35% hydrogen peroxide (Whiteness HP, FGM) and randomly assigned to G1 artificial saliva, G2 2% sodium fluoride (Flugel, Nova DFL), G3 casein phosphopeptide-amorphous calcium phosphate fluoride paste (CPP-ACPF, MI Paste Plus, GC America), G4 rinse for color maintenance after bleaching (Keep White Rinse, DMC) and G5 polishing with aluminum oxide-impregnated disks (Super Buff Disk, Shofu). Fifty specimens were immersed in red wine for 15 minutes, immediately after treatment, and the others one hour after. Color difference (∆E) was evaluated with a spectrophotometer (Vita EasyShade). Surface treatments and waiting time effects were analyzed with Kruskal-Wallis and Mann Whitney tests (*p*<0.05).

**Results:**

Surface treatments (*p*>0.05) and waiting time (*p*>0.05) were not significant to decrease bleached enamel susceptibility to red wine staining.

**Conclusions:**

Surface treatments were similar to artificial saliva for bleached enamel susceptibility to red wine staining. Immediate or one-hour-postponed contact with red wine did not affect bleached enamel color.

** Key words:**Tooth bleaching, color, dental enamel, hydrogen peroxide, pigmentation.

## Introduction

Direct extrinsic staining agents include dietary components and behavioural agents, as red wine and smoking. The organic chromogens are adsorped onto acquired pellicle, and the final color is determined by natural chromogen color (1,2). The chromogen is usually incorporated into biofilm (2).

Bleached dental enamel can be more susceptible to staining than the non bleached (1,3-6), especially right after tooth bleaching (3,4,7). Superficial roughness increases after bleaching and this enhances teeth staining by dye adhesion (8,9). This consequence is more pronounced with colourant food and beverage intake (10). A possible reason for deletary effects on enamel is the bleaching gel pH. The surface roughness increases with pure 35% hydrogen peroxide but not with a mixture of sodium bicarbonate (NaHCO3) and 30% hydrogen peroxide, which was attributed to the bigger pH of the mixture, compared to the more acid pH of 35% hydrogen peroxide (11). Colourant beverages with acid pH may cause enamel mineral loss, modify the surface and reduce the resistance to staining after bleaching (12).

Instrumental analysis offers a potential advantage on visual determination of color, since device reading can be quantified (13). The CIE system of colorimetry is based on primary colors (X, Y and Z) and the functions of color combinations for each wave-length (14). The ΔE=1 is the minor difference of color perceived by a device and ΔE≤3,3 is considered acceptable (15).

Fluoride and other remineralizing solutions can favor a positive balance toward remineralization (7). The complex casein phosphopeptide-amorphous calcium phosphate (CPP-ACP) induces less staining immediately after bleaching (7). Casein, calcium and phosphate are responsible for the resistance to acid dissolution (16). When the CPP-ACP or the casein phosphopeptide-amorphous calcium phosphate fluoride (CPP-ACPF) are applied, the reactive part CPP quickly bonds to the biofilm, depositing calcium and phosphate ions where they are needed. Calcium and phosphate free ions debond of CPP, penetrate into enamel rods and regenerate apatite crystals (16).

The waiting times of 30 and 150 minutes after bleaching are not different considering coffee and red wine immersion, and a significant staining is induced by with red wine, not by coffee (17). The waiting time of 15 minutes for red wine still was not evaluated.

This study aimed to evaluate the effect of different surface treatments and waiting time prior to contact with dye on bleached enamel staining susceptibility. The null hypothesis is that there is no difference on color regarding waiting time nor regarding tested surface treatments.

## Material and Methods

This *in vitro* study has a randomized complete block design. Independent variables were surface treatments and waiting time before contact with dye (immediate or one-hour postponed). One hundred bovine dental crowns were randomly divided in 10 complete blocks. The dependent variable was the color difference (∆E), evaluated with a spectrophotometer (n=10).

Sample calculation was performed according to a pilot study (n=4):

Mean ∆E in control group =9.0

Mean ∆E in treatment group =14.35

Standard deviation of the variable =3.96

Standardized magnitude of effect =(14.35–9.0/3.96)=1.35 Considering bilateral α 0.05 and β 0.80 a simplified formula for Student t test was applied (18): N=16/( Standardized magnitude of effect)2=16/1.352 =8.8

Minimum sample size estimated was 9 teeth, but 10% was added, to compensate possible losses (=10 teeth/group).

One hundred bovine incisors were stored in distilled water (6±1°C) for one week. The teeth were sectioned on the cementum-enamel junction, and the pulps were extracted under irrigation with 2.5% sodium hypochlorite. They were cleaned with an ultrasound device, and pumice.

The crowns were examined with an optical microscope (8x) to exclude specimens with surface defects. The pulp chamber entrance was sealed with zinc oxide-eugenol paste (Lysanda Produtos Odontológicos, São Paulo, Brazil). A 6mm2 area was delimited with an adhesive tape on the incisal third of each buccal surface, and two layers of nail polish (Risqué Niasi S.A., Taboão da Serra, Brazil) were applied. Then, the adhesive tape was removed to expose the experimental area. Specimens were identified and stored in distilled water (6 ± 1°C) for 24 hours.

The first reading with the digital spectrophotometer (Vita Easyshade Compact, Vita, Bad Säckingen, Germany) was performed. The spectrophotometer tip was in contact perpendicularly with the experimental area. The device was calibrated in the beginning of the study and after evaluating each specimen. The color readings were registered according to the CIEL*a*b* tridimensional system. Three readings of L*, a* and b* were performed for each sample, and the mean was calculated.

The specimens were bleached with 35% hydrogen peroxide (Whiteness HP, LOT 170214, FGM Produtos Odontológicos, Joinville, Brazil) with 18 drops of hydrogen peroxide and 6 drops of thickener. Bleaching agent pH was measured with a potentiometer (Metrohm 827 pH lab, Metrohm Pensalab Instrumentação Analítica LTDA, São Paulo, Brazil) and ranged from 5.49 to 5.40 within 10 to 15 minutes. Three applications of the bleaching agent were performed (15 minutes each), simulating one bleaching session without application of light. The specimens were washed and subjected to a second evaluation of color.

Specimens were divided into five groups according to the randomized complete block design. A spreadsheet for randomization generated with Microsoft Excel (Microsoft Corporation, Redmond, USA) was applied.

Group 1 (G1): Immersion in neutral artificial saliva for 10 minutes, which contained 0.96g KCL, 0.67g NaCl, 0.04g MgCl2, 0.27g monobasic potassium phosphate, 0.12g calcium phosphate tricalcium, 10ml preservative solution, 24ml 70% sorbitol, 8g carboxymethylcellulose and bidistilled water to bring the volume to a final 1000ml. The preservative solution formula was 15% Nipagin, 5% Nipasol and propylene glycol.

Group 2 (G2): Neutral 2% sodium fluoride (Flugel, LOT 15050588, DFL Indústria e Comércio S.A. Rio de Janeiro, RJ, Brazil) for 4 minutes.

Group 3 (G3): Casein phosphopeptide-amorphous calcium phosphate fluoride (CPP-ACPF) paste (GC MI Paste Plus™, LOT 120411M, GC Corporation, Tokyo, Japan) for 3 minutes.

Group 4 (G4): Two sprays of a rinse for maintenance of bleaching results (Keep White Rinse, LOT 20615, DMC, São Carlos, Brazil).

Group 5 (G5): Polishing with aluminum oxide-impregnated feltrum disks (Super Buff, LOT 0411711, Shofu, San Marcos, USA) for 10 seconds.

After surface treatments, G2, G3, G4 and G5 were rinsed for 10 seconds.

Five specimens of each block were immersed in 25ml of red wine (Canção Bordô Suave Serra Gaúcha, Antonio Basso e filhos LTDA, Flores da Cunha, Brazil), immediately after surface treatment (Time 0), and the other five after one hour after (Time 1). During waiting time, they were maintained in artificial saliva. They were stained for 15 minutes in an incubator at 37±1oC and cleaned with a brush in low-speed handpiece and dentifrice (Colgate Total 12, Colgate-Palmolive Company, New York, USA) for 3 seconds. Then, they were washed and subjected to the 3rd color evaluation.

**Figure 1:**
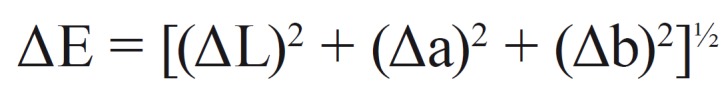
Formula.

The differences of L* (ΔL), a* (Δa) and b* (Δb) were calculated using the values obtained during the baseline evaluation, after bleaching and after staining. The color parameters ΔLb, Δab and Δbb were obtained by the difference between baseline and after bleaching; and the color parameters ΔLs, Δas and Δbs by the difference between after bleaching and after staining. To calculate the total difference in color obtained with bleaching (ΔEb) and with staining (ΔEs), this formula was applied (5,7,9-11,13-15), (Fig. 1):

The smaller the ΔE, smaller is the difference of color between the evaluated phases.

Normal distribution and homogeneity of variance of data were analyzed with Kolmogorov Smirnov and Levene. The effect of surface treatments and waiting time on color was analyzed with Kruskal-Wallis and Mann Whitney. The confidence level applied on the statistical software SPSS 17 (Statistical Product and Service Solutions, SPSS, Chicago, USA) was 5%.

## Results

With Kruskal-Wallis test, there was no significant difference between surface treatments on bleached enamel susceptibility to staining with red wine in T0 and T1 (*p*>0.05) ( Table 1).

**Table 1:**
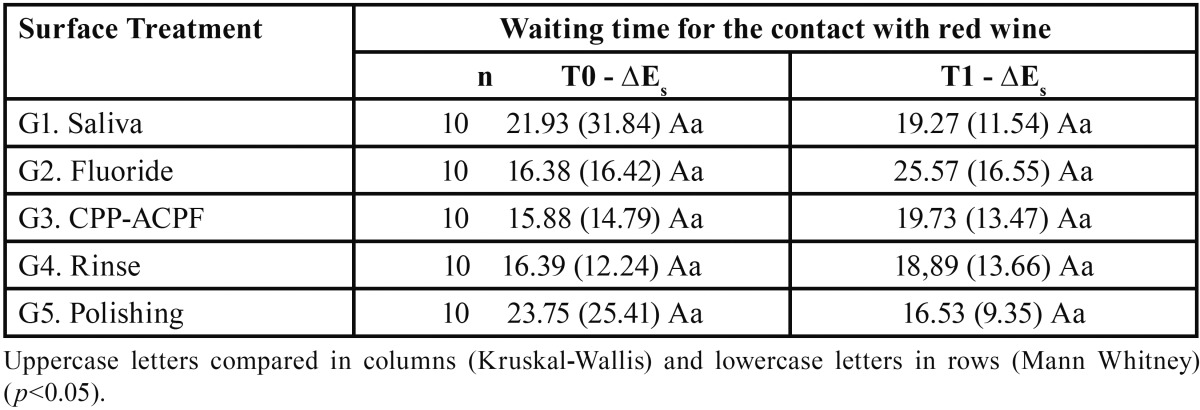
Medians (interquartile range) of the color difference of bleached enamel subjected to different surface treatments and to red wine staining (∆Es), in T0 and T1.

With Mann-Whitney test, there was no significant difference between T0 and T1 on bleached enamel susceptibility to staining with red wine in all groups (*p*>0.05) ( Table 1).

## Discussion

The bleaching effect decreases in part after one month, and then it remains more stable (15); there is an increase in staining due to consumption of beverages even on 10% carbamide peroxide bleached teeth (10). A rough enamel, with irregularities after bleaching stains more easily (1,8) since food dyes can adhere to rough surface (9). Resultant erosion depths are different with bleaching agents of different pHs, with approximately 0.27µm for 30% H2O2-NaHCO3 (higher pH) and 0.85µm for 35% H2O2 (more acid) (11). Considering that bleaching enhances the susceptibility to staining (5), it becomes necessary to find treatments to minimize it.

For enamel color alteration and mineral loss investigation, enamel blocks of G1 were stored in artificial saliva for 3 weeks; G2 received 10% carbamide peroxide for 6 hours/day and artificial saliva between the bleaching sessions. Specimens of G3, G4 and G5 received the same as G2, but after bleaching they were immersed for one hour in cola soft drink, melted chocolate and red wine, respectively. Specimens of G3 and G5 showed higher mineral loss. Specimens of G5 showed the highest color alteration while G1 the lowest. Staining food and beverages of acid pH can induce mineral loss on enamel, modify the surface and reduce the resistance to staining after bleaching (12).

In our study, there was no difference between the effects of fluoride, CPP-ACPF, keep white rinse and polishing when compared to control, stored in artificial saliva. When comparing to similar studies we should consider the influence of the bleaching agent, as its pH can change enamel surface. However, even using the same agent, neutral fluoride effect after bleaching may be different with other staining methods (6). Instrumental analysis of color minimizes the bias of visual determination, since color can be quantified (13) with CIELAB system. It applies a non linear transformation of XYZ values to L*, a* and b* coordinates, in a tridimensional space of color, where a* and b* axes form a plane, and L* axis is orthogonal (14).

Enamel susceptibility to staining was investigated in different waiting times after 35% hydrogen peroxide bleaching. There was a control group, a group stained in coffee 30 minutes after bleaching, a group stained in coffee after 150 minutes, a group stained in red wine after 30 minutes, and a group stained in red wine after 150 minutes. There was no difference between the waiting times for both beverages. Bleached enamel was susceptible to staining with red wine on both waiting times after bleaching, while coffee did not influence bleaching proccess (17).

In our study, waiting one hour for specimens immersion in red wine on staining susceptibility of bleached enamel was statistically similar to immediate staining after the surface treatments. We may infer that it is not necessary to wait to ingest coloured beverages after bleaching since there is no positive effect on color maintenance, although some reports found higher susceptibility to staining immediately after bleaching (3,4,7).

Bleached enamel microstructural defects can be repaired by absorption and precipitation of saliva components, such as calcium and phosphate (7). Although saliva has some potential for remineralization, it cannot increase available calcium and phosphate levels by itself (16). However, the potential for remineralization *in vivo* could neutralize the adverse effects of bleaching, such as calcium loss (2).

There is a remineralizing effect of artificial saliva, fluoride and CPP-ACP solution, which prevent more effectively dyes absorption in 24 hours after surface treatment than in one hour (7). Considering our study findings, saliva could have a protective effect on bleached enamel, therefore inducing the same surface treatments effects, probably remineralizing. However, since we did not use natural saliva, our model has some limitations due to artificial saliva composition and its effects, in spite of the constant effort to approximate this *in vitro* model to the reality.

Other studies are necessary to investigate saliva properties and its effects on the decrease of bleached enamel staining susceptibility, in addition to elucidate what are the differences between artificial and natural saliva on this proccess. With this study, we may conclude that the effects of surface treatments with neutral sodium fluoride, CPP-ACPF, keep white rinse and polishing with aluminum oxide impregnated feltrum disks after tooth bleaching were similar to that induced by artificial saliva regarding bleached enamel susceptibility to red wine staining. Immediate or one-hour-postponed contact with red wine did not affect bleached enamel color.
